# Comparative Outcomes of Pars Plana Vitrectomy With and Without Adjunct Laser Photocoagulation in Optic Disc Pit Maculopathy

**DOI:** 10.1155/joph/3889015

**Published:** 2026-05-05

**Authors:** Mehmet Sahin Sevim, Semra Sevim

**Affiliations:** ^1^ Departments of Ophthalmology, World Eye Hospital, Altunizade Branch, Istanbul, Turkey; ^2^ Departments of Ophthalmology, Haydarpasa Numune Training and Research Hospital, Istanbul, Turkey, haydarpasanumune.gov.tr

**Keywords:** anatomical success, endolaser photocoagulation, gas tamponade, optic disc pit maculopathy, pars plana vitrectomy

## Abstract

**Purpose:**

Optic disc pit maculopathy (ODP‐M) is a rare condition that can lead to progressive visual impairment. Although pars plana vitrectomy (PPV) has become the mainstay of treatment, the additional benefit of adjunct peripapillary laser photocoagulation remains controversial. We hypothesized that adjunct laser photocoagulation does not provide additional anatomical or functional benefit when combined with PPV and gas tamponade. This study aimed to compare the anatomical and functional outcomes of PPV with and without adjunct peripapillary endolaser photocoagulation in the management of ODP‐M.

**Methods:**

This retrospective comparative study included 19 eyes of 17 patients who underwent PPV with gas tamponade for ODP‐M between January 2013 and August 2024. Patients were divided into two groups according to whether endolaser photocoagulation was applied to the temporal margin of the optic disc during surgery. Pre‐ and postoperative best‐corrected visual acuity (BCVA), central retinal thickness (CRT), and anatomical outcomes were compared between groups. Anatomical success was defined as complete resolution of subretinal and/or intraretinal fluid on spectral‐domain optical coherence tomography (SD‐OCT).

**Results:**

The mean postoperative follow‐up duration was 20 months (range, 12–36 months). Anatomical success was achieved in 9 of 10 eyes (90%) in the laser group and 8 of 9 eyes (89%) in the nonlaser group (*p* = 1.00). The mean postoperative BCVA was 0.40 ± 0.27 logMAR and 0.41 ± 0.24 logMAR, respectively (*p* = 0.82). Both groups showed significant visual improvement compared to baseline, but there were no intergroup differences in BCVA or CRT changes. No intraoperative or postoperative complications such as retinal detachment, macular hole formation, or endophthalmitis were observed.

**Conclusion:**

PPV with gas tamponade provides favorable anatomical and functional outcomes in ODP‐M. The addition of endolaser photocoagulation does not yield further benefit and may be unnecessary in standard PPV‐based management.

## 1. Introduction

Optic disc pit (ODP) is a rare congenital excavation of the optic nerve head that can lead to the accumulation of intraretinal and subretinal fluid in the macular area, resulting in optic disc pit maculopathy (ODP‐M) and progressive visual impairment [1, 2]. The incidence of ODP is approximately 1 in 10,000, and the condition is typically unilateral, though bilateral involvement occurs in up to 15% of cases [3]. The exact mechanism of macular detachment remains uncertain, but vitreous traction and a potential communication between the subarachnoid and intraretinal spaces have been proposed [4–8].

Over the past 2 decades, pars plana vitrectomy (PPV) has become the mainstay of treatment for ODP‐M [9]. The surgical rationale involves relieving vitreoretinal traction, inducing posterior vitreous detachment [10], and sealing or reducing fluid communication through internal limiting membrane (ILM) manipulation and intraocular gas tamponade [11, 12]. Adjunctive techniques such as peripapillary laser photocoagulation, ILM flap “plugging,” and macular buckling have also been described, yet no clear consensus exists on the optimal approach [13–15].

Peripapillary laser photocoagulation has historically been applied along the temporal margin of the optic disc to promote chorioretinal adhesion and limit fluid migration toward the macula [16]. In clinical practice, low‐intensity focal argon or diode laser photocoagulation applied in a limited peripapillary pattern is generally preferred in order to minimize collateral retinal damage. However, several recent studies have questioned its necessity when vitrectomy with ILM peeling and gas tamponade is performed, reporting comparable anatomical outcomes without laser application. Moreover, concerns regarding potential damage to the retinal nerve fiber layer have led many surgeons to abandon routine laser use [17–19].

The present study aimed to compare the anatomical and functional outcomes of PPV with and without adjunct peripapillary laser photocoagulation in patients with ODP‐M. The primary objective of the study was to compare anatomical success between the two groups. Secondary objectives included comparison of postoperative visual improvement, changes in central retinal thickness (CRT), mean fluid resolution time, and postoperative complications.

## 2. Patients and Methods

This retrospective, comparative study included 19 eyes of 17 patients who underwent PPV with gas tamponade for ODP‐M between January 2013 and August 2024 at Haydarpaşa Numune Training and Research Hospital and World Eye Hospital, Altunizade Branch. All surgeries were performed by the same surgeon (M.S.S.). The patients were divided into two groups according to whether endolaser photocoagulation was applied to the temporal margin of the optic disc during surgery. In both groups, patients with clinically and optical coherence tomography (OCT)–confirmed ODP and associated maculopathy were included. The group treated without adjunct laser photocoagulation was considered the control group, while the group receiving additional endolaser photocoagulation represented the intervention group. This retrospective observational study was conducted and reported in accordance with the Strengthening the Reporting of Observational Studies in Epidemiology (STROBE) guidelines.

Eligibility criteria were defined as follows. Inclusion criteria were eyes with clinically and OCT‐confirmed ODP‐M treated with PPV and gas tamponade, with a minimum follow‐up of 12 months. Exclusion criteria included eyes with other retinal diseases that could affect macular structure or visual acuity (such as diabetic retinopathy, retinal vein occlusion, or age‐related macular degeneration), previous vitreoretinal surgery, or insufficient follow‐up data.

**FIGURE 1:**
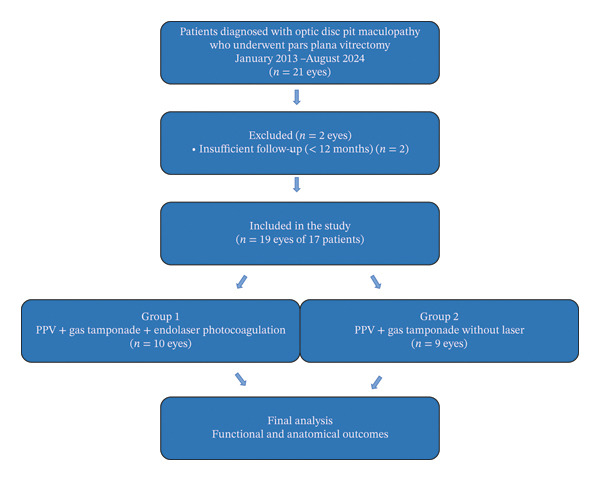
Flow diagram illustrating patient selection and group allocation in the study comparing pars plana vitrectomy with and without adjunct endolaser photocoagulation for optic disc pit maculopathy.

Pre‐ and postoperative best‐corrected visual acuity (BCVA), duration of symptoms, preoperative CRT measured by OCT, the presence of outer retinal schisis or macular hole, and postoperative anatomical status (attached vs. detached macula) were evaluated. Macular OCT imaging was performed preoperatively and at postoperative 1, 3, 6, and 12 months. Only patients with a minimum follow‐up of 12 months were included. Snellen visual acuity values were converted to logarithm of the minimum angle of resolution (logMAR) equivalents for statistical analysis. The patient selection and group allocation process is illustrated in Figure 1.

The surgical procedure consisted of a standard three‐port 23‐ or 25‐gauge PPV. After core vitrectomy, posterior hyaloid detachment was induced using triamcinolone acetonide assistance in eyes without spontaneous separation. No additional macular surgical maneuvers, such as ILM peeling, were performed in any case. In Group 1, endolaser photocoagulation was applied as a single row of burns along the temporal margin of the optic disc using low‐intensity settings (power: 200 mW, duration: 100 ms) in order to minimize potential retinal nerve fiber layer damage. The decision to apply adjunct endolaser photocoagulation was based on the surgeon’s intraoperative preference and evolving surgical practice during the study period.

At the end of all surgeries, meticulous peripheral retinal inspection was performed to detect any iatrogenic breaks.

Postoperatively, patients were prescribed topical prednisolone acetate and moxifloxacin hydrochloride four times daily. The topical antibiotic was discontinued after 3 days, while the steroid was tapered weekly and discontinued after 1 month.

All patients were examined on postoperative day 1 and at 1, 3, 6, and 12 months. Additional visits were scheduled as necessary. Final BCVA and anatomical success were recorded after the 12‐month follow‐up.

Statistical analyses were performed using IBM SPSS Statistics Version 28.0 (IBM Corp., Armonk, NY, USA). Continuous variables were expressed as mean ± standard deviation (SD) or median [minimum–maximum], and categorical variables were expressed as number and percentage (%). Normality of distribution was assessed using the Shapiro–Wilk test. Intergroup comparisons of continuous variables were performed using the independent samples *t*‐test for normally distributed data and the Mann–Whitney *U* test for nonnormally distributed data. Categorical variables were compared using the chi‐square test or Fisher’s exact test when expected cell counts were below five. Changes in BCVA and CRT were further analyzed using analysis of covariance (ANCOVA), with preoperative values entered as covariates. A *p* value of < 0.05 was considered statistically significant. Because of the relatively small sample size, multivariate regression or propensity score matching analyses were not performed, as such approaches would not provide statistically reliable estimates in this cohort.

## 3. Results

A total of 19 eyes from 17 patients diagnosed with ODP‐M were included in the study. Ten eyes underwent adjunct endolaser photocoagulation (Group 1), while nine eyes did not (Group 2). The mean postoperative follow‐up duration was 20 months (range, 12–36 months).

The demographic and clinical characteristics of the patients are summarized in Table 1. Baseline BCVA was comparable between groups. The mean preoperative BCVA was 0.16 ± 0.10 logMAR (approximately 20/30 Snellen) in the laser group and 0.15 ± 0.08 logMAR (approximately 20/30 Snellen) in the nonlaser group. The mean age was similar between the two groups (Group 1: 32.80 ± 8.13 years; Group 2: 34.11 ± 10.53 years; *p* = 0.77). No statistically significant differences were observed between groups regarding sex distribution, preoperative symptom duration, baseline BCVA, or CRT (*p* > 0.05). The presence of outer retinal schisis, splitting, or small holes at baseline was also comparable between the two groups.

**TABLE 1:** Baseline characteristics.

Variable	Group I (laser+) (*n* = 10)	Group II (laser−) (*n* = 9)	*p* value
Age (years)	32.80 ± 8.13	34.11 ± 10.53	0.77
Sex (F/M)	6/4	4/5	—
Symptom duration (months)	17.40 ± 16.43	20.44 ± 14.85	0.68
Preop BCVA (logMAR)	0.16 ± 0.10	0.15 ± 0.08	0.75
Preop CRT (*µ*m)	807.00 ± 105.41	826.67 ± 143.35	0.74
Outer retinal changes (yes/no)	5/5	4/5	1.00

*Note:* Data are presented as mean ± SD unless otherwise indicated.

Abbreviations: BCVA, best‐corrected visual acuity; CRT, central retinal thickness.

## 4. Functional Outcomes

Both groups demonstrated a significant improvement in BCVA at the final follow‐up compared with preoperative values. The mean postoperative BCVA was 0.40 ± 0.27 logMAR (approximately 20/50 Snellen) in the laser group and 0.41 ± 0.24 logMAR (approximately 20/50 Snellen) in the nonlaser group (*p* = 0.82). The mean change in BCVA (ΔBCVA) was 0.24 ± 0.22 logMAR and 0.26 ± 0.22 logMAR, respectively, with no statistically significant intergroup difference (*p* = 0.82) (Table 2). Overall, the majority of eyes (approximately two‐thirds) achieved a visual gain of ≥ 0.3 logMAR, and adjunct laser photocoagulation did not produce a significant difference in functional outcomes.

**TABLE 2:** Surgical outcomes.

Outcome	Group I (laser+) (*n* = 10)	Group II (laser−) (*n* = 9)	*p* value
Postop BCVA (logMAR)	0.40 ± 0.27	0.41 ± 0.24	0.93
BCVA change (logMAR)	0.24 ± 0.22	0.26 ± 0.22	0.82
Anatomic success (yes/no)	9/10	8/9	1.00
Fluid resolution time	7.4 ± 2.8	7.7 ± 3.0	0.84

*Note:* No statistically significant differences were found between groups (*p* > 0.05 for all comparisons).

Abbreviation: BCVA: best‐corrected visual acuity.

### 4.1. Anatomical Outcomes

Anatomical success, defined as complete resolution of subretinal and/or intraretinal fluid on spectral‐domain optical coherence tomography (SD‐OCT), was achieved in 9 of 10 eyes (90%) in Group 1 and 8 of 9 eyes (89%) in Group 2 (*p* = 1.00, Fisher’s exact test) (Table 2). No recurrence of macular detachment was observed in any patient during the follow‐up period. The mean time to resolution of intraretinal and/or subretinal fluid was 7.4 ± 2.8 months in the laser group and 7.7 ± 3.0 months in the nonlaser group. No statistically significant difference was observed between the two groups (*p* > 0.05).

### 4.2. Complications

No intraoperative or postoperative complications such as retinal detachment, macular hole formation, endophthalmitis, or sustained intraocular pressure elevation were observed in either group. Progression of cataract was noted in some phakic eyes and was managed with routine phacoemulsification during the follow‐up period.

## 5. Discussion

This study compared the anatomical and functional outcomes of PPV with or without adjunct endolaser photocoagulation in the treatment of ODP‐M. Our findings demonstrated comparable anatomical resolution and visual improvement in both groups, indicating that endolaser photocoagulation did not confer additional benefit. These results are consistent with previous reports highlighting PPV as the mainstay of treatment for ODP‐M [2, 20].

Rayat et al. [17] reported complete foveal reattachment in 81% of 32 eyes and a mean visual gain of five Snellen lines following PPV, with no significant additive effect from endolaser or ILM peeling. Similarly, Avcı et al. [18] achieved 100% anatomical success and 85% visual improvement in 13 eyes treated with PPV and gas tamponade without ILM peeling. These outcomes align closely with the 89%–90% anatomical success rate observed in our series. The absence of significant differences in anatomical or visual outcomes between the laser and nonlaser groups supports the adequacy of PPV alone in achieving satisfactory results.

The purpose of endolaser photocoagulation is to limit fluid communication between the optic pit and the macula by promoting chorioretinal adhesion [1]. However, recent studies have shown that posterior hyaloid detachment and gas tamponade performed during PPV are sufficient to achieve this effect, making adjunct laser unnecessary [16]. Iyer et al. [21] reported that laser treatment did not significantly improve anatomical success and may carry a potential risk of retinal nerve fiber layer damage. Similarly, in the meta‐analysis by Meng et al. [22], the overall anatomical success rate after PPV was approximately 85%, with no significant improvement when laser or ILM peeling was added. Recent studies have suggested that defects within the retinal nerve fiber layer or papillomacular bundle may represent an alternative pathway for intraretinal fluid migration in ODP‐M. In such cases, laser photocoagulation applied to the temporal margin of the optic disc may not effectively block this pathway, which could partly explain the limited additional benefit of laser treatment observed in our study [23, 24].

Although some centers suggest that laser may reduce recurrence of intraretinal or subretinal fluid, most authors agree that it is not a decisive factor for anatomical recovery. Studies by Hirakata et al. and Theodossiadis et al. demonstrated that PPV without laser yields stable long‐term anatomical outcomes [20]. In our series, no recurrence of macular detachment was observed over a mean follow‐up of 20 months. Furthermore, the absence of complications in the nonlaser group supports the view that endolaser is not required for safety considerations.

Another point of debate in ODP‐M management is the role of ILM peeling. While several authors have suggested that ILM peeling may accelerate fluid resorption, it also carries potential risks [21, 22]. In our study, satisfactory anatomical and functional outcomes were achieved without ILM peeling, suggesting that a minimally invasive approach may be sufficient. Nevertheless, recent meta‐analyses indicate that techniques such as ILM flap or pit‐plugging may be beneficial in selected cases, although these methods are not yet standardized [22].

One important limitation of the present study is the relatively small sample size, which may have reduced the statistical power to detect subtle differences between groups. In addition, the use of ANCOVA in small cohorts should be interpreted with caution, as the estimates may be less stable and potentially influenced by residual confounding.

Although alternative statistical approaches such as permutation tests, sensitivity analyses, or matching techniques may be considered, these methods were not deemed appropriate given the limited sample size and the risk of overfitting or further reduction in effective sample size. Larger prospective studies are required to allow the application of more robust statistical methodologies.

Recent advances in high‐resolution imaging have enhanced understanding of ODP‐M pathogenesis, emphasizing the roles of vitreoretinal traction and incomplete posterior hyaloid separation [21]. Accordingly, PPV with relief of vitreoretinal traction, gas tamponade–induced subretinal fluid resorption, and closure of potential pit–macula communication appear sufficient to restore anatomy and function. Consistent with this mechanism, our study achieved anatomical success without the need for additional laser treatment.

In conclusion, PPV combined with posterior hyaloid detachment and gas tamponade appears adequate to achieve both anatomical and functional recovery in ODP‐M. Adjunct endolaser photocoagulation does not provide additional benefit and may carry potential risks. Larger, prospective studies are warranted to confirm these findings. From a clinical perspective, these findings suggest that PPV with gas tamponade alone may be sufficient for the management of ODP‐M, and routine adjunct laser photocoagulation may not be necessary in standard surgical practice.

## Funding

This research received no specific grant from any funding agency in the public, commercial, or not‐for‐profit sectors.

## Ethics Statement

This retrospective study was conducted in accordance with the principles of the Declaration of Helsinki. Institutional review board approval was waived because of the retrospective design and the use of anonymized patient data. Written informed consent was obtained from all patients for the surgical procedure and for the use of anonymized clinical data in this publication.

## Conflicts of Interest

The authors declare no conflicts of interest.

## Data Availability

The data that support the findings of this study are available from the corresponding author upon reasonable request.
